# PCSK9 Inhibitor: Safe Alternative to Fill the Treatment Gap in Statin-Limited Conditions?

**DOI:** 10.31083/j.rcm2311380

**Published:** 2022-11-09

**Authors:** Ying Xiao, Zhengqing Ba, Shurui Pang, Dong Liu, Hao Wang, Hanyang Liang, Yong Wang, Jiansong Yuan

**Affiliations:** ^1^Fuwai Hospital, National Center for Cardiovascular Diseases, Chinese Academy of Medical Sciences & Peking Union Medical College, 100037 Beijing, China; ^2^Institute of Basic Medical Sciences, Chinese Academy of Medical Sciences & Peking Union Medical College, 100730 Beijing, China; ^3^Peking Union Medical College Hospital, Chinese Academy of Medical Sciences & Peking Union Medical College, 100730 Beijing, China

**Keywords:** cardiovascular disease, anti-PCSK9 antibody, proprotein convertase subtilisin/kexin type 9 inhibitors, statin, adverse effects, hypolipidemic therapy

## Abstract

Lipid-lowering therapy is of great importance in reducing the burden of atherosclerotic cardiovascular disease. Statins act as first-line therapy in the current lipid management guidelines. However, statin use is limited in (1) statin-induced adverse events, including statin-associated muscle symptoms, new-onset diabetes mellitus, drug-induced liver injuries, acute kidney injuries, cognitive effects, hemorrhagic strokes, and cataracts; (2) special populations, including pregnant and lactating patients, patients with decompensated cirrhosis, and patients on dialysis; (3) coadministration with statin-interactive drugs, such as anti-human immunodeficiency virus drugs, anti-hepatitis C virus drugs, and immunosuppressive drugs. These considerable statin-limited groups are in urgent need of safer alternative lipid-lowering options. Proprotein convertase subtilisin/kexin type 9 (PCSK9) inhibitors are attracting widespread attention for their documented safety in general populations and superior lipid-lowering properties. Therefore, questions have been raised whether PCSK9 inhibitors could be a safe alternative in patients who are intolerant to statin therapy. In this review, we discuss the safety of PCSK9 inhibitors in statin-limited conditions. We conclude that PCSK9 inhibitors are a safe alternative lipid-lowering therapy in various statin-limited conditions. Furthermore, we identify several limitations in the current literature and suggest future directions, for the refinement of lipid management regimens.

## 1. Introduction 

Cardiovascular (CV) diseases, of which atherosclerotic cardiovascular disease (ASCVD) is the major component, are the leading cause of death worldwide, accounting for one-third of all causes of death [1]. Dyslipidemia is the dominant risk factor for ASCVD [2]. Statins are recognized as first-line lipid-lowering therapy for managing dyslipidemia [2, 3].

Statins have been shown to be an efficacious and safe lipid-lowering therapy in the majority of patients with dyslipidemia [4, 5]. However, real-world data reveals a divergence from guideline recommendations. Only one-third (31.9%) of patients with severe hypercholesterolemia and a low-density lipoprotein cholesterol (LDL-C) ≥4.9 mmol/L received guideline-recommended statin therapy, according to the 2021 updated report from the American Heart Association (AHA) [1]. Meanwhile, up to 50% of patients receiving statin therapy showed poor adherence [6]. This poor utilization and adherence of statins are largely due to various safety concerns, including statin-induced adverse events (AEs), restrictions in specific populations, and statin-drug interactions [6, 7].

Unfortunately, no established consensus guidelines currently exist regarding alternative non-statin lipid-lowering therapy (LLT) in these statin-limited groups [2, 3]. Consequently, there is an urgent need for a safe alternative in patients who are intolerant to statin therapy.

Proprotein convertase subtilisin/kexin type 9 (PCSK9) inhibitor, a newly discovered lipid-lowering agent, is thought to be a potential “Game Changer” for lipid management. PCSK9 inhibitors have demonstrated to be the most powerful lipid-lowering drug that is currently available to treat hypercholesterolemia [8, 9, 10, 11, 12, 13, 14, 15]. Evolocumab and alirocumab, two PCSK9 inhibitors currently approved by the US Food and Drug Administration (FDA), can significantly reduce LDL-C levels by approximately 60%, whether as add-on therapy or monotherapy [16, 17]. In addition, PCSK9 inhibitors been shown to be safe in all groups of patients. Adverse Events (AEs) of PCSK9 inhibitors are infrequent and include inject-site reactions, myalgia, and flu-like symptoms, most of which are mild [13, 14, 16, 17]. Therefore, PCSK9 inhibitors have emerged as an alternative to statins and have become a new lipid-lowering option in recent years.

Currently, there has been no comprehensive review focusing on the safety of PCSK9 inhibitors in statin-limited groups. Therefore, our review sought to answer the following questions: (1) What are the limitations of statins? (2) Are PCSK9 inhibitors a safe alternative when statins are limited? (3) What prevents the widespread use of PCSK9 inhibitors?

## 2. Search Strategy

This review article was based on a systematic search conducted in PUBMED and the Cochrane Library. Hand searching was also used to find relevant studies in PubMed and other websites (e.g., FDA and ICER). In addition, international guidelines on cardiovascular disease (CVD) prevention and lipid management were included [2, 3]. The search strategies addressed the following concepts: statins, adverse effects, drug toxicity, drug tolerance, drug interactions, drug contraindications, and PCSK9 inhibitors. Articles published in English between 2000 and 2022 were included. Two authors conducted the screening and selection process independently, and a consensus was reached in all instances.

## 3. Clinical Pharmacology

The comparison of pharmacokinetic properties between statins and PCSK9 inhibitors is listed in Table 1 (Ref. [18, 19]).

**Table 1.** **Comparison of pharmacokinetic properties between statins and PCSK9 inhibitors [18, 19]**.

Drug	Administration	t1/2	Bioavailability	CYP, OATP, P-gp substrate	Elimination
Statins	Oral	0.5–30 h	5–30% (Pitavastatin >60%)	YES	Fecal excretion 60–90%;
					Urinary excretion 0–20%
PCSK9 inhibitors	Injection	11–20 d	72–85%	NO	Receptor-mediated endocytosis (at low concentration);
					Non-saturable proteolytic pathway (at high concentration)

t1/2, drug half-time; CYP, cytochrome P450; OATP, organic anion transporting polypeptide; P-gp, P-glycoprotein.

### 3.1 Statins

Currently, there are seven statins approved by the FDA: lovastatin, simvastatin, pravastatin, fluvastatin, atorvastatin, rosuvastatin, and pitavastatin [18].

The lipid-lowering effect of statins is mediated by the competitive inhibition of 3-hydroxy-3-methylglutaryl–coenzyme A (HMG-CoA) reductase, a rate-limiting step in hepatic endogenous cholesterol synthesis. Statin therapy has been shown to decrease LDL-C levels by 20–50%, triglyceride (TG) levels by 10–20%, and cause an increase in high-density lipoprotein cholesterol (HDL-C) levels by 1–10% [3, 19]. Apart from the lipid-lowering effect, statins have also been found to have pleiotropic effects which result in anti-inflammatory, anti-oxidant, anti-thrombotic, and procalcifying properties [2, 3, 20, 21].

All statins are absorbed rapidly after oral administration. The liver is the major site of statin metabolism and elimination. Statins are absorbed from the portal vein into hepatocytes through membrane transporters OATP1B1 (organic anion transporting polypeptide 1B1) and OATP1B3 (organic anion transporting polypeptide 1B3). Lipophilic statins (simvastatin, lovastatin, atorvastatin, fluvastatin, pitavastatin) can also be absorbed into hepatocytes through passive diffusion. In the liver, statins are predominately metabolized by cytochrome P450 (CYP450) isoenzymes (except for pravastatin). Simvastatin, lovastatin, and atorvastatin are metabolized through the CYP3A4 isoenzyme, while fluvastatin, pitavastatin, and rosuvastatin are metabolized through the CYP2C9 isoenzyme. The liver biotransformation of statins account for their low bioavailability. Statins are eliminated via the bile into the feces or urine. The hepatic elimination of statins accounts for the majority of statin elimination, mediated by multiple drug membrane transporters, including OATP1B1 and P-glycoprotein (P-gp). Renal elimination accounts for only a small proportion of statin elimination (<20%) [19, 20].

### 3.2 PCSK9 Inhibitors

Currently, there are two PCSK9 inhibitors approved by the FDA: alirocumab and evolocumab, both of which are human immunoglobulin G (IgG) monoclonal antibodies (mAbs) [16, 17].

PCSK9 is produced by hepatocytes and is present in plasma. It binds to the LDL receptor on the cell membrane and results in lysosomal degradation of the receptor, resulting in decreased LDL-C clearance and increased serum LDL-C levels. PCSK9 inhibitors can inhibit the binding of PCSK9 to LDLR by binding to serum PCSK9, thereby decreasing LDL receptor degradation and increasing LDL-C uptake, and ultimately decreasing serum LDL-C levels by approximately 60%. PCSK9 inhibitors can also decrease TG levels by 8–10% and increase HDL-C levels by 8–10%. PCSK9 inhibitor is the only approved lipid-lowering drug that can significantly reduce lipoprotein a (Lp (a)) level by 30–40% [3, 22]. In addition to its excellent lipid-lowering effects, the anti-inflammatory effect of PCSK9 inhibitors has also been noted in recent studies [23, 24, 25].

Evolocumab and alirocumab are subcutaneously administered, with a high absolute bioavailability of 72% and 85%. Due to the biochemical characteristics of mAbs, PCSK9 inhibitors do not affect the CYP450 enzymes and membrane transporters (e.g., OATP1B1 and P-gp) that metabolize and eliminate statins. Instead, they degrade into small peptides and individual amino acids. The elimination of PCSK9 inhibitors undergoes two phases: at low concentrations through saturable binding to PCSK9 and at high concentrations through a non-saturable proteolytic pathway [16, 17, 26, 27].

## 4. Safety of PCSK9 Inhibitors in Statin-Induced AEs 

**Fig. 1.**
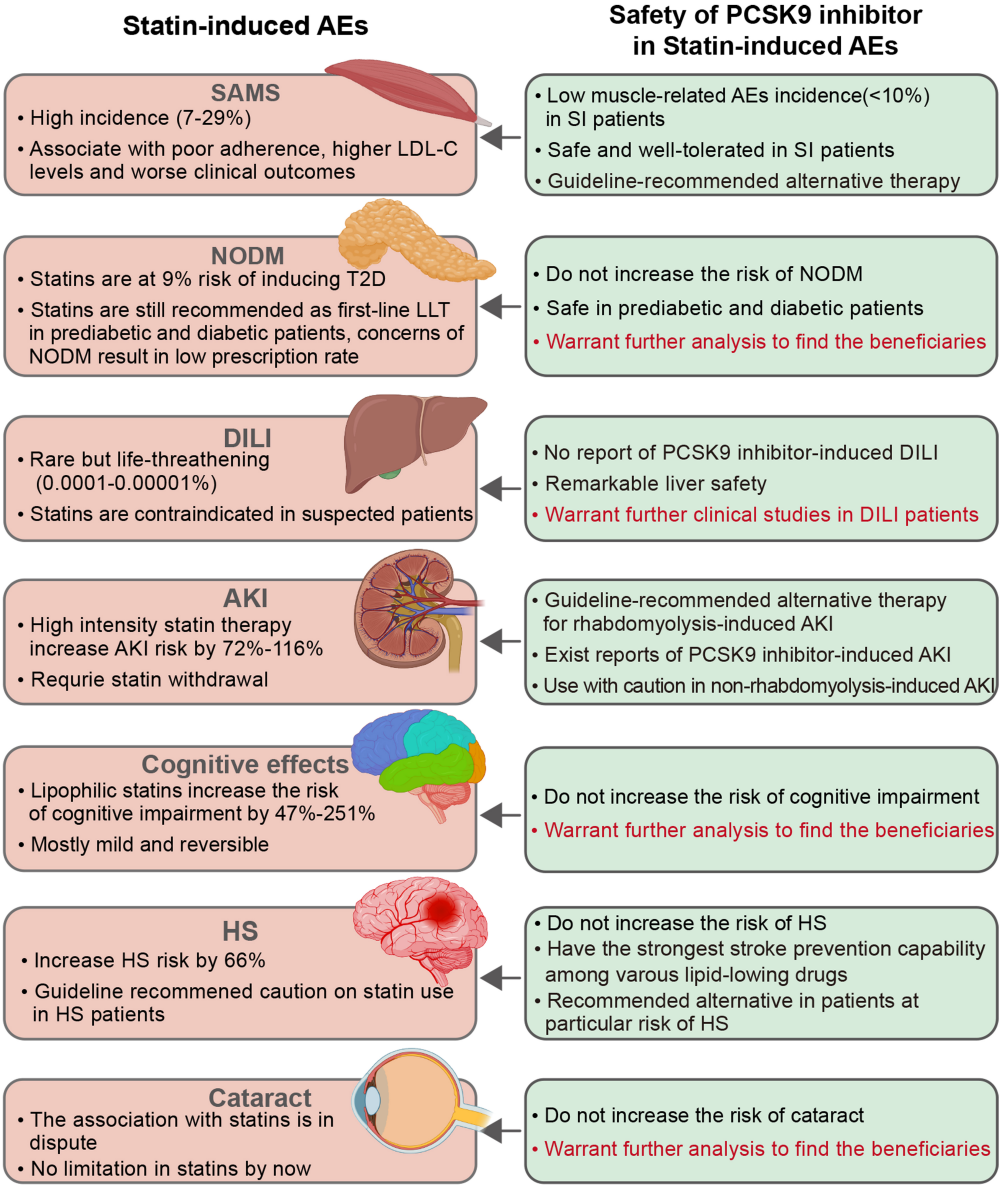
**Safety of PCSK9 inhibitors in statin-induced AEs**. Words in red color outline the future research directions of PCSK9 inhibitors as statin alternative therapy. PCSK9, proprotein convertase subtilisin/kexin type 9; AE, adverse event; SAMS, statin-associated muscle symptoms; SI, statin intolerance; LDL-C, low-density lipoprotein cholesterol; NODM, new-onset diabetes mellitus; T2D, type 2 diabetes; LLT, lipid-lowering therapy; DILI, drug-induced liver injury; AKI, acute kidney injury; HS, hemorrhagic stroke.

The safety of PCSK9 inhibitors in statin-induced AEs are summarized in Fig. 1.

### 4.1 Statin-Associated Muscle Symptoms (SAMS)

#### 4.1.1 Statin Status

The National Lipid Association proposed the most recognized definition of statin intolerance (SI) in 2014, defining SI as a clinical syndrome incapable of tolerating at least two statins (one at the lowest starting daily dose and another at any daily dose) due to either objectionable syndromes (subjective or objective) or abnormal laboratory results, which occurred upon exposure to statin treatment, and is reversible after statin discontinuation and reproducible by statin rechallenge [28].

Statin-associated muscle symptoms (SAMS) are responsible for the vast majority of SI. Hence, most studies and guidelines, including our review, equate SAMS with SI [2, 3, 29]. SAMS are characterized by myalgias, and weakness with or without elevated serum muscle enzymes [26], and ranges from 7–29% among all patients on statin therapy and has become the predominant cause for discontinuation in 62% of patients [5, 30, 31].

A retrospective cohort study of 20,760 patients demonstrated that SAMS contributes to poor LDL-C goal attainment (OR = 1.85; [95% CI: 1.60–2.16]; *p *< 0.0001), higher medical costs (cost ratio = 1.20; [95% CI: 1.11–1.28]; *p *< 0.0001), and a higher risk for revascularization procedures compared with control groups [32]. Females, the elderly, patients with concurrent conditions (e.g., liver or renal dysfunction, diabetes), or concomitant interacting medications (e.g., anti-HIV, anti-HCV, immunosuppressive drugs) are at higher risk for developing SAMS [33, 34, 35].

Several large RCTs on statins have reported much lower rates of SAMS than observational studies, based on which “nocebo” effect has been proposed [36]. Some of the muscle symptoms of statins may be caused by the patients’ psychological expectations of harm from treatment rather than by the drug itself. At present, there is no effective method to distinguish between the patients who develop muscle symptoms due to the “nocebo” effect and the statin itself [37]; and a double-blind study is neither practical or ethical.

In the significant number of statin users who exhibit muscle symptoms in the clinical setting, traditional solutions have been to adjust statin therapy by dose reduction/intermittent dosing, or changing to another statin. Despite these efforts, about 30% of these patients still cannot tolerate statins [38]. Furthermore, many non-statin lipid lowing drugs, such as ezetimibe, bile acid sequestrants, fibrates, and niacin, have significant limitations. Their lipid lowering properties are limited, and they have a high incidence of AEs, thereby questioning their qualifications as an alternative LLT [39].

#### 4.1.2 Safety of PCSK9 Inhibitors in SAMS

PCSK9 inhibitors are associated with a low incidence of muscle-related AEs (generally <10%). Myalgia is the most common form of PCSK9 inhibitor-induced muscle-related AEs. In addition, PCSK9 inhibitors have a favorable safety and tolerability profile in SI patients in a series of large clinical trials (Table 2, Ref. [8, 9, 10, 11, 12, 15]).

**Table 2.** **Summary of key randomized controlled trials on PCSK9 inhibitors in SI patients**.

PCSK9 inhibitor	Trial name	Study Population	Median follow-up	Intervention	Safety Results	Reduction in plasma LDL-C level, %
Evolocumab	GAUSS [8]	SI (n = 160)	12-week	(1) 280, 350, or 420 mg q4w	Myalgia was the most common treatment-emergent AEs, 280 mg:15.6% (n = 5); 350 mg: 3.2% (n = 1); 420 mg: 3.1% (n = 1); 420 mg and ezetimibe: 20.0% (n = 6); placebo and ezetimibe: 3.1% (n = 1).	280 mg: 40.8% (95% CI: 32.9%–48.6%); 350 mg: 42.6% (95% CI: 34.7%–50.5%); 420 mg: 50.7% (95% CI: 42.8%–58.6%)
				(2) 420 mg q4w and Ezetimibe 10 mg		
				(3) placebo q4w and Ezetimibe 10 mg		
	GAUSS-2 [9]	SI; Not reach LDL-C goal (n = 307)	12-week	(1) 140 mg q2w	(1) Muscle-related AEs rates were evolocumab: 12% (n = 25); ezetimibe: 23% (n = 23).	140 mg q2w: 56.1% (95% CI: 52.5%–59.7%); 420 mg q4w: 55.3% (95% CI: 52.3%–58.3%)
				(2) 420 mg q4w	(2) Myalgia was the most common treatment-emergent AEs, evolocumab: 8 (n = 16); ezetimibe: 18% (n = 18).	
				(3) Ezetimibe 10 mg	(3) AE-induced discontinuation rate were evolocumab: 8% (n = 17); ezetimibe: 13% (n = 13).	
	GAUSS-3 [10]	SI; Not reach LDL-C goal (n = 511)	24-${\mathrm{week}}^{1}$	(1) 420 mg q4w	(1) Muscle-related AEs occurred in 20.7% (n = 30) of evolocumab group and 28.8% (n = 21) of ezetimibe group.	54.5% (95% CI: 51.8%–57.2%)
				(2) Ezetimibe 10mg	(2) Muscle symptom-induced discontinuation rate were evolocumab: 8.3% (n = 12); ezetimibe: 6.8% (n = 5).	
	OSLER-1 [15]	Subjects from phase 2 evolocumab trials (n = 1324)	4-year	(1) 420 mg q4w	(1) The annualized AE rate of muscle-related AEs was 4.7% in the evolocumab group and 8.5% in the placebo group.	61% (95% CI: 60%–63%)
				(2) placebo	(2) Noted a trend that reports of new-onset muscle-related AEs decreased with increasing years of drug exposure.	
Alirocumab	ODYSSEY ALTER-NATIVE [11]	SI; at moderate-to-high CV risk (n = 361)	24-week	(1) 75/150 mg q2w	Alirocumab group was at a lower rate of muscle-related AEs compared with atorvastatin (HR: 0.61; 95% CI: 0.38–0.99; *p* = 1.042) and showed a trend toward a lower rate compared with ezetimibe (HR: 0.71; 95% CI 0.47–1.06; *p* = 0.096)^2^.	54.8%
				(2) Atorvastatin 20 mg		
				(3) Ezetimibe 10 mg		
	ODYSSEY MONO [12]	LDL-C 100–190 mg/dL; 10-year risk of fatal CV events 1%–5%	24-week	(1) 75mg q2w	Muscle-related AEs occurred in 3.8% (n = 2) of evolocumab group and 3.8% (n = 2) of ezetimibe	54.1%
				(2) ezetimibe 10 mg	

LDL-C, low-density lipoprotein cholesterol; SI, statin intolerance; AE, adverse event; CV, Cardiovascular; HR, hazard ratio; CI, confidence interval. ^1^ refers to phase B, in this phase evolocumab was introduced. ^2^ reduction in plasma LDL-C level at a mean of weeks 10 and 12.

Based on the above studies, PCSK9 inhibitors are now incorporated in the latest updated guidelines as an acceptable alternative therapy for patients with SI [2, 3]. A real-world cohort study in the Netherlands indicated that among patients prescribed PCSK9 inhibitors, 42.9% (n = 102) were SAMS. SAMS has become the leading indication for PCSK9 inhibitor therapy [29]. Most current studies of PCSK9 inhibitors in SAMS patients are short-term follow-up trials (the longest 4 years) [11]. Therefore, the long-term safety of PCSK9 inhibitors as an alternative to statins remains to be studied.

In conclusion, since SAMS has become a common issue associated with poor clinical outcomes, current studies suggest PCSK9 inhibitors are a safe and generally well-tolerated alternative LLT for SAMS patients [8, 9, 10, 11, 12, 15]. Additional well-designed long-term clinical trials are required to confirm the long-term safety of PCSK9 inhibitors’ monotherapy.

### 4.2 New-Onset Diabetes Mellitus (NODM)

#### 4.2.1 Statin Status

In March 2012, the FDA updated statin labeling and added concerns about the risk of statin-induced elevated blood glucose levels and new-onset diabetes mellitus (NODM) [16]. This update is based on well-designed clinical studies and epidemiological data, indicating a 9% non-negligible risk of statin-induced Type 2 diabetes (T2D) in patients who received statins [40, 41, 42]. The mechanisms of statin-induced NODM are not fully understood. Multiple mechanisms, including pancreatic β-cell dysfunction, peripheral insulin resistance, and modulation of microRNAs expression, have been found to contribute to statins’ diabetogenic effects [43].

Statins are still recommended as the first line LLT for patients with high to very-high CV risk by international guidelines regardless of the risk of developing NODM [2, 3, 44]. This is because the benefits of LDL-C reduction on CVD prevention exceed the absolute risk of the incidence of NODM. In a meta-analysis including 5 statin trials with 32752 patients, intensive statin therapy accounted for 2 additional cases of NODM per 1000 patient-years, whereas 6.5 cases fewer CVD events were observed [45].

Unfortunately, real-world data revealed a gap between therapeutic guidelines and clinical treatment. Among 72136 patients with diabetes at very-high CV risk in Italy, 35% did not receive statin therapy, while only 15% of those patients receiving statin therapy reached the guideline-recommended LDL-C level (LDL-C <1.4 mmol/L) [35]. In addition to concerns about the effect of statins on plasma glucose, diabetes as a risk factor for SAMS, is also thought to be a reason for this low prescription rate [35]. These issues highlight the need for improvement in LLT for these patients.

#### 4.2.2 Safety of PCSK9 Inhibitors in NODM

Although genetic data suggest that patients carrying loss-of-functionPCSK9 genetic variants are at higher risk of developing T2D (OR = 1.29; [95% CI: 1.11–1.50]) [46], results in clinical trials and meta-analysis are at variance with the genetic studies and found that PCSK9 inhibitors do not increase the risk of NODM [47, 48, 49].

A prespecified analysis of the Further Cardiovascular Outcomes Research With PCSK9 Inhibition in Subjects With Elevated Risk (FOURIER) trial included 11,031 patients with diabetes and 16,533 patients without diabetes at baseline, and demonstrated that evolocumab neither increases the risk of NODM in patients without diabetes at baseline (8.0% versus 7.6%; HR = 1.05; [95% CI: 0.94–1.17]) nor in patients with prediabetes at baseline (11.3% versus 11.2%; HR = 1.00; [95% CI: 0.89–1.13]) during a median follow-up of 2.2 years. Evolocumab also showed no influence on glycemic control. Levels of glycosylated hemoglobin Type A1C (HbA1c) and fasting plasma glucose were similar between the evolocumab and placebo groups [47]. Similarly, a pooled analysis of 10 ODYSSEY Phase 3 trials (n = 4974) showed no evidence of alirocumab-induced NODM compared with placebo- (HR = 0.90; [95% CI: 0.63–1.29]) and ezetimibe- (HR = 1.10; [95% CI: 0.57–2.12]) controlled groups during a follow-up period of 6–18 months [48]. Moreover, these two studies showed a similar incidence of AEs between the evolocumab/alirocumab and placebo groups.

The inconsistent outcomes in NODM risk between genetic findings and clinical trials of PCSK9 inhibitors may be attributed to two factors: (1) PCSK9 inhibitor treatment has a shorter duration on PCSK9 concentrations compared with genetic variants. (2) mAb mainly targets circulating PCSK9s. Hence, unlike genetic variants that affect both intracellular and extracellular PCSK9 concentrations, mAbs have a limited effect on intracellular PCSK9 concentrations in pancreatic beta cells, resulting in an insignificant effect on glycemia [47, 48].

In summary, statins still play an important role in the LLT of prediabetic and diabetic patients. However, since PCSK9 inhibitors do not affect blood glucose levels and do not increase the incidence of NODM, additional studies are necessary to determine those patients who will be the beneficiaries of alternative PCSK9 inhibitors therapy among prediabetic/diabetic patients with dyslipidemia.

### 4.3 Drug-Induced Liver Injury (DILI)

#### 4.3.1 Statin Status

Drug-induced liver injury (DILI) refers to a rare (0.0001–0.00001% for any single medication) but life-threatening type of hepatitis, which is the most common etiology for acute hepatic failure. 75% of patients with DILI result in mortality or liver transplantation [50]. Data between 1994 and 2012 from the Spanish Hepatotoxicity Registry showed that 5.5% (n = 47) of DILI were attributable to statins [51].

All statins have been reported to trigger DILI [51]. The mechanism of statin-induced DILI remains unclear. Yu, Geng, *et al*. [52] indicated that drugs metabolized by CYP450 enzymes (including all FDA-approved stains except for pravastatin) had a 4 fold higher possibility (OR = 3.99; [95% CI: 2.07–7.67]) of causing DILI compared with placebos, and were significant predictors for DILI (OR = 5.04; [95% CI: 2.34–10.9]).

Therefore, liver function tests are recommended before initiation of statin therapy. Unexplained persistent elevation of serum transaminases (suggesting the possibility of DILI) and acute liver diseases are FDA contraindications for statins [18].

#### 4.3.2 Safety of PCSK9 Inhibitors in DILI

Unlike statins and other lipid-lowering drugs, PCSK9 inhibitors appear to be relatively free of any significant hepatotoxicity and have little influence on liver function [53, 54]. No case of PCSK9 inhibitor-induced DILI has been reported. Consequently, neither monitoring of liver enzymes nor limitations on the status of a patient’s liver function are mentioned on PCSK9 inhibitors’ labels [16, 17].

The resumption of statin therapy is prohibited in patients with stain-induced DILI and patients who have experienced a severe liver injury after re-exposure to another statin [18, 55]. Therefore, PCSK9 inhibitors offer a promising option for alternative LLT after statin-induced DILI., but will need to be confirmed in large cohort and case-crossover studies.

### 4.4 Acute Kidney Injury (AKI)

#### 4.4.1 Statin Status

AKI results in increased mortality and high healthcare costs. Patients with CVD are at higher risk for AKI [56].

Statins have been shown to induce AKI. There are currently three known mechanisms involved in statin-induced AKI [56, 57, 58]: direct or immune-mediated kidney interstitial injury, rhabdomyolysis-induced AKI, and suppression of coenzyme Q10. A nationwide cohort study in France which included 8,236,279 participants demonstrated that high-potency statin treatment was associated with a 72%–116% increased risk of AKI (HR = 1.72; [95% CI: 1.37–2.17] in men, and HR = 2.16; [95% CI: 1.64–2.85] in women) [56]. Therefore, statins should be immediately discontinued in patients with statin-induced AKI.

#### 4.4.2 Safety of PCSK9 Inhibitors in AKI

PCSK9 inhibitors are a guideline-recommended alternative LLT for patients with SAMS, including rhabdomyolysis-induced AKI [2, 3]. Dardano, Daniele *et al*. [59] reported that an 80-year-old male developed rhabdomyolysis-induced AKI under rosuvastatin therapy, and that ezetimibe failed to effectively reduce LDL-C levels after statin withdrawal (LDL-C: 5.9–6.6 mmol/L). Therefore alirocumab was initiated, and LDL-C levels remained under control (1.2 mmol/L) with no AEs during the 24-month follow-up period [59].

Although PCSK9 inhibitors are a safe alternative for rhabdomyolysis-induced AKI patients, unexplained alirocumab-induced acute tubular injuries have been reported in two studies [60, 61]. Although these are the only two reports of PCSK9 inhibitors-induced renal injury, the potential for nephrotoxicity needs to be monitored when considering PCSK9 inhibitors as an alternative in non-rhabdomyolysis-induced AKI.

### 4.5 Cognitive Effects

#### 4.5.1 Statin Status

The cognitive effects of statins in literature are highly inconsistent, reporting beneficial, detrimental, and no effects, keeping this topic in a heated debate until now.

An analysis of the FDA Adverse Event Reporting System demonstrated an increased risk of cognitive impairment among statin users, especially in lipophilic statins (proportional reporting ratios range: 1.47–3.51 in lipophilic statins versus 0.69–1.64 in hydrophilic statins) [62]. The possible mechanism responsible for this phenomenon is that lipophilic statins cross the blood-brain barrier to a greater extent than hydrophilic statins, lowering brain cholesterol levels and inducing myelin impairment, ultimately leading to cognitive impairment [63]. Moreover, statin-induced suppression of coenzyme Q10 may enhance oxidative stress, reduce cerebral energy production and contribute to cognitive dysfunction [63].

Nevertheless, several recent clinical studies and meta-analyses have shown no significant effect of statins on cognitive functions [64, 65]. A randomized trial of 18,846 patients ≥65 years old revealed no significant association between statins and dementia and other mild cognitive impairments compared with the non-statin group [64]. Furthermore, Schultz, B. G. *et al*. [63] proposed that the lipid-lowering effect of statins can result in beneficial cognitive effects by protecting cerebral vessels and reducing Aβ formation, and preventing dementia and Alzheimer’s disease. However, the Cochrane Systematic Review of 26,340 participants found no protective effects of statins on dementia (including Alzheimer’s disease) compared with placebo groups [65].

Based on these controversial results and the lack of consensus, there have been no strict limitations on statin use regarding cognitive impairments. In 2012, the FDA released a safety warning about the possible adverse cognitive side effects (such as memory loss and confusion) induced by statins. This warning was updated in 2016, describing these memory impairments as notable but non-serious, reversible after statin discontinuation, and more likely to happen in patients over 50 [18].

#### 4.5.2 Safety of PCSK9 Inhibitors on Cognitive Effects

Current studies show no significant association between PCSK9 inhibitors and cognitive impairments. The EBBINGHAUS (Evaluating PCSK9 Binding Antibody Influence on Cognitive Health in High Cardiovascular Risk Subjects) study evaluated cognitive function in 1974 patients from the FOURIER study and found no significant difference between evolocumab and placebo groups during a median follow-up of 19 months [66]. No impairment in cognitive function is inserted in the labels of alirocumab and evolocumab [16, 17].

Given the ability of PCSK9 inhibitors to reduce serum LDL-C to extremely low levels, there are questions as to whether this could contribute to brain cholesterol deficiency and impair cognitive function [63].

Reassuringly, large clinical trials such as the FOURIER and the ODYSSEY OUTCOMES trials demonstrated that alirocumab- or evolocumab-induced low LDL-C levels resulted in no significant differences concerning cognitive AEs compared with placebo groups [13, 14]. This most likely due to the relatively large volume which disables mAbs from directly crossing the blood-brain barrier [66].

In conclusion, current studies suggest PCSK9 inhibitor treatment has little effect on cognitive function. Nevertheless, given that most reports of cognitive impairment in statin users are mild and reversible [18, 67], further studies are needed to determine if patients can benefit from PCSK9 inhibitors as an alternative in terms of cognitive safety.

### 4.6 Hemorrhagic Stroke (HS)

#### 4.6.1 Statin Status

Statins have been shown to reduce the risk of ischemic stroke but increase the risk of hemorrhagic stroke (HS), which is less prevalent but more likely to be fatal.

This conclusion is mainly based on the SPARCL trial, a large-scale randomized study including 4731 patients with a previous stroke or transient ischemic attack. This study demonstrated that 80 mg of atorvastatin per day increased the risk of HS significantly by 66% (adjusted HR = 1.66; [95% CI: 1.08–2.55]) with a median follow-up period of 4.9 years [68]. This potential hemorrhagic propensity is thought to be derived from statins’ (not lipid-lowering mediated) pleiotropic effects, such as anti-thrombotic properties that decreases platelet aggregation and thrombogenesis [21].

Based on these findings, current clinical guidelines on stroke management recommend caution on statin use in patients with previous spontaneous intracerebral hemorrhage (the most common form of HS), especially before introducing a high-dose statin regimen [69].

#### 4.6.2 Safety of PCSK9 Inhibitors in HS

Unlike statin’s anti-thrombotic ability, PCSK9 inhibitors were found to reduce the risk of ischemic stroke while having no association with HS (RR = 0.93; 95% CI: 0.58–1.51) in a meta-analysis involving 5 PCSK9 inhibitor RCTs (76,140 patients) [21].

In regard to safety concerns that achieving extremely low LDL-C levels might increase the risk of stroke, recent studies did not support this hypothesis and showed a consistent risk-reducing effect of lowing LDL-C levels on all strokes [21, 70]. Each 1 mmol/L decrease in LDL-C level was associated with a significant reduction of all stroke risk by 23.5% (slope = 0.235; [95% CI: 0.007–0.464]). PCSK9 inhibitors are proven to have the greatest ability to reduce stroke rates among various lipid-lowering drugs [70].

In view of these data, there is a call for consideration about to use PCSK9 inhibitors, instead of high-intensity statins, in patients at particular risk of HS [70]. In order to justify this proposal, the comparisons between PCSK9 inhibitor monotherapy and statins on the risk of HS should be further studied in well-designed large clinical trials.

### 4.7 Cataract

#### 4.7.1 Statin Status

Cataract, defined as loss of lens transparency, accounts for the leading cause of blindness worldwide [71].

There is controversy concerning the association between cataracts and statin treatment. Cataracts developed in animal experiments in dogs given lovastatin and simvastatin in dosages well above the maximal clinical doses [72]. A propensity score-matched analysis involving 46,249 subjects from a US administrative dataset demonstrated that statin users are at higher risk of cataracts compared with nonusers (adjusted OR = 1.25; [95% CI: 1.14–1.38]), and statins acted as an independent predictor for the development of cataracts (adjusted OR = 1.43; [95% CI: 1.33–1.53]) [73]. The mechanism of statin-induced cataracts might be due to long-term inhibition of the de novo synthesis of cholesterol in the lens that can result in lens opacity. Moreover, the bidirectional effects of statins on oxidative stress may also contribute to this process [73].

On the other hand, several studies produced inconsistent results, reporting no effect or protective effect of statins on cataracts [71]. Since there is no clear conclusion, no limitation of statins in patients with or at risk of cataracts is currently indicated.

However, given the widespread prescription of statins and the irreversible loss of vision caused by cataracts, the risk of statin-induced cataracts among the elderly (old age is the most critical risk factor for cataract formation) and long-term users should not be ignored.

#### 4.7.2 Safety of PCSK9 Inhibitors in Cataracts

PCSK9 inhibitors can achieve lower LDL-C levels than statins, while no side effect leading to cataracts has been detected. A meta-analysis including 5 trials with 83,492 patients demonstrated that PCSK9 inhibitor treatment was not associated with increased cataract risk (OR = 0.96; [95% CI: 0.85–1.08]) [74]. The analysis of VigiBase found no disproportionality between PCSK9 inhibitors and cataracts (reporting odds ratios = 0.64; [95% CI: 0.48–0.85]) [75].

This phenomenon might occur because PCSK9 inhibitors could play a lipid-lowering role without inhibiting endogenous cholesterol synthesis, the dominant cholesterol synthesis pathway in the lens [75].

Hence, PCSK9 inhibitors appear to be a safer option in patients at risk for cataracts. However, it is essential to underline that there are limited studies of PCSK9 inhibitors on this issue. Therefore, coupled with statins’ unconfirmed cataract-induced capability, no definitive conclusion can be made until further investigations are performed.

## 5. Safety of PCSK9 Inhibitors in Special Populations

**Fig. 2.**
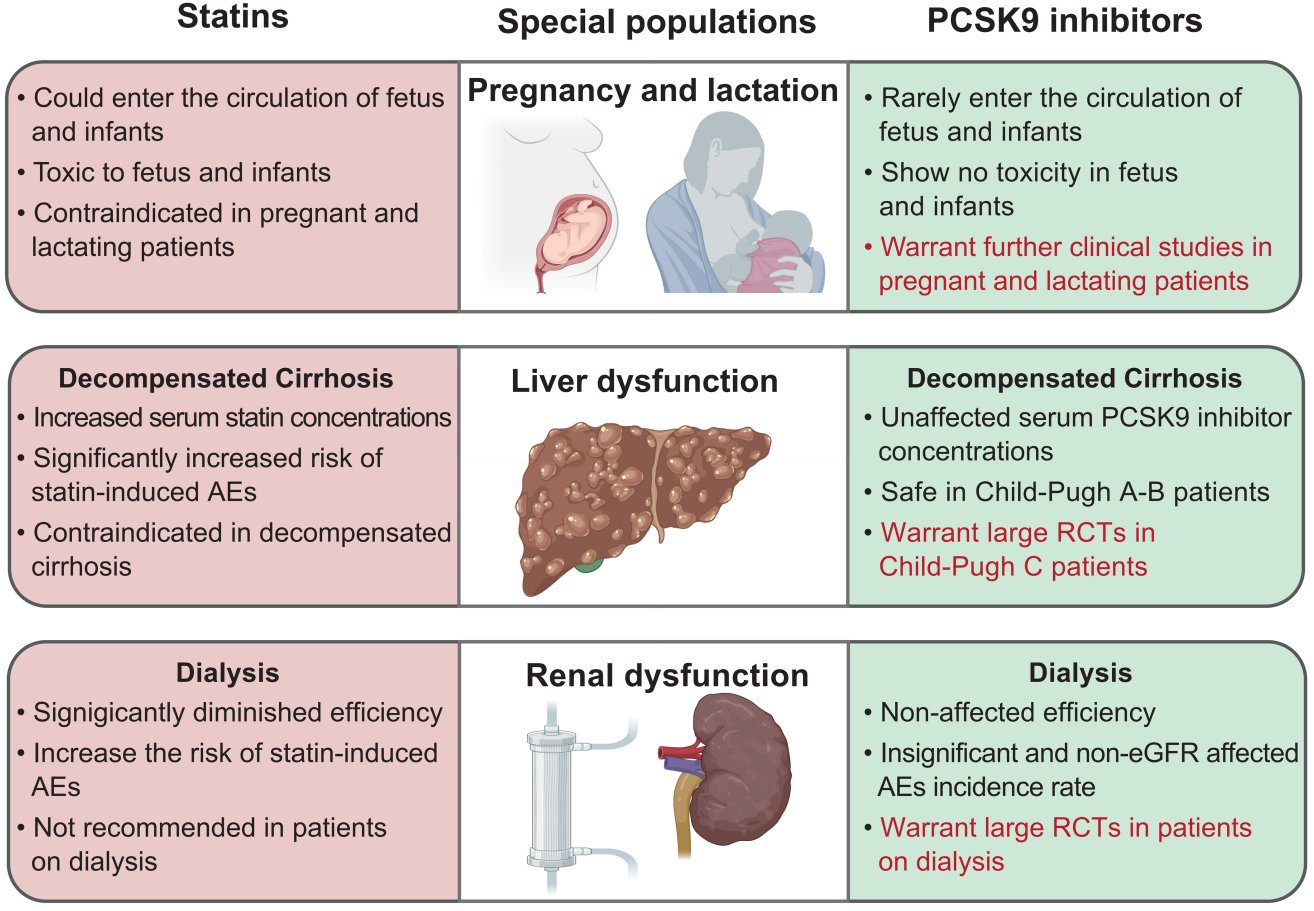
**Safety comparisons between statins and PCSK9 inhibitors in special populations**. Words in red color outline the future research directions of PCSK9 inhibitors as statin alternative therapy. PCSK9, proprotein convertase subtilisin/kexin type 9; AE, adverse event; eGFR, estimated glomerular filtration rate; RCT, randomized controlled trial.

The safety comparisons between statins and PCSK9 inhibitors in special populations are summarized in Fig. 2.

### 5.1 Pregnancy and Lactation

#### 5.1.1 Statins Status

Dyslipidemia in pregnancy is associated with an increased risk of AEs such as pre-eclampsia, pregnancy-induced hypertension, and gestational diabetes [76].

Nevertheless, statins are recommended to be discontinued in patients with pregnancy and lactation according to the latest update of the FDA in 2021. The mechanism of statins’ underlying fetal toxicity remains unclear, and may related to the following mechanisms [18, 77, 78, 79, 80]: Cholesterol and other products of the cholesterol biosynthesis pathway play an essential role in fetal growth and development. Statins can cross the placental barrier or pass into breast milk and cause harm to the fetus. Higher abortion rates and teratogenicity have been observed in animals (rats, mice, rabbits) exposed to high-intensity statins [77, 78]. A retrospective cohort study compared 281 statin-exposed pregnant women to 2643 controls and reported a higher rate of spontaneous miscarriage in the statin-exposed group (25.27% versus 20.81%; adjusted HR = 1.64; [95% CI: 1.1–2.46]) [79]. Currently, the option of LLT is strictly limited in pregnant and lactating patients.

#### 5.1.2 PCSK9 Inhibitors in Pregnancy and Lactation

There are no restriction in the labels of PCSK9 inhibitors regarding patients in pregnancy or lactation [16, 17].

The most significant potential advantages of PCSK9 inhibitors lie in the safety of mAbs in pregnant and lactating patients. First mAbs do not cross the placenta in early pregnancy. Animal experiments (rats, monkeys) demonstrated no adverse effects on embryo-fetal development despite being given concentrations tens of times higher than the maximum recommended human dose [81]. Second, current FDA-approved PCSK9 inhibitors are both IgG mAbs, which are present in breast milk but do not enter the circulation of newborns and infants in significant amounts via breastfeeding [16, 17, 26].

The current findings suggest PCSK9 inhibitors are expected to be a potential emerging therapy for pregnant and lactating patients and dyslipidemia with contraindications to statins. However, due to the lack of population-based studies, it is still in the preliminary exploration stage. At present, a prospective observational study of evolocumab on patients during pregnancy is in progress and will provide us with further data [82].

### 5.2 Liver Dysfunction

#### 5.2.1 Relationship with the Liver

The comparison of statins and PCSK9 inhibitors in the relationship with the liver is listed in Table 3.

**Table 3.** **Comparison of statins and PCSK9 inhibitors in the relationship with the liver**.

Relationship with the liver	Statins	PCSK9 inhibitors
Lipid lowing mechanism	inhibiting a key step in hepatic cholesterol synthesis (HMG-CoA reductase)	binding to serum PCSK9
Liver metabolism and elimination	YES	NO
Transaminases elevation	YES	NO
DILI	YES	NO

HMG-CoA, 3-hydroxy-3-methylglutaryl–coenzyme A; DILI, drug-induced liver injury; PCSK9, proprotein convertase subtilisin/kexin type 9.

5.2.1.1 Statins

The liver is the primary site of the lipid-lowering effect of statins and their metabolism and elimination. Therefore, liver dysfunction can attribute to elevated statin concentrations and increase the risk of statin-induced AEs. On the other hand, statins also display a non-negligible effect on liver function [19]. Although the specific mechanism remains unclear, a transient moderate level (less than 3X the upper limit of normal elevation) of serum transaminases can be observed after stain intake, and 0.5–3.0% of patients had persistent increases (to more than 3X the upper limit of normal elevation) in serum transaminases [55]. Concerns for statins’ hepatotoxicity and reports of DILI (discussed in the DILI section) limits the application of statin therapy in patients with liver dysfunction [18].

5.2.1.2 PCSK9 Inhibitors

The metabolism and elimination of PCSK9 inhibitors do not rely on hepatic function [16, 17]. Among PCSK9 inhibitors users, the current literature shows no elevation (RR = 0.94; [95% CI: 0.84–1.06]) [53] or slight elevation (a mean increase of 5.8 mg/dL in ALT and 6.2 mg/dL in AST, respectively) [54] in serum transaminases, which appeared to be clinically insignificant. Generally, PCSK9 inhibitors showed better liver safety profiles compared with other lipid-lowering drugs [83]. Furthermore, given recent studies showing that serum PCSK9s are involved in the pathogenesis of hepatic steatosis and alcohol-induced inflammation [23, 24], PCSK9 inhibitors are increasingly utilized and expected to play a protective role in liver diseases.

#### 5.2.2 Decompensated Cirrhosis

5.2.2.1 Statin Status

Decompensated cirrhosis is the progressive period of end-stage chronic liver disease. Although CVDs appear in more than 1/3 of patients with cirrhosis and have become an important reason for mortality, lipid management remains challenging in this population. Severely impaired liver function magnifies the hepatotoxicity of medications [84]. The National Lipid Association’s Statin Liver Safety Task Force lists decompensated cirrhosis as a contraindication for statin therapy [34].

The severe hepatic impairment and portal-systemic shunting during the decompensated cirrhosis phase significantly interferes with multiple statin metabolism and elimination processes, leading to high serum statin concentrations and increasing the risk of statin-induced AEs [85, 86]. A meta-analysis that included 24 studies demonstrated a 40-fold increased risk of rhabdomyolysis (4% versus 0.01%) among cirrhosis patients taking simvastatin 40 mg compared with the general population [85].

5.2.2.2 Safety of PCSK9 Inhibitors in Decompensated Cirrhosis

Although the data in severe hepatic impaired (Child-Pugh C) patients is not available, the recommended dosages of PCSK9 inhibitors (75 mg/mL for evolocumab and 140 mg/mL for alirocumab) have been proven to be safe in mild-to-moderate hepatic impaired patients (Child-Pugh A-B) [16, 17, 53, 87].

Recent findings of improvements in hepatic steatosis and alcohol-induced inflammation after inhibition of PCSK9 suggest PCSK9 inhibitors will become a novel therapeutic target for non-alcoholic fatty liver disease and alcoholic liver disease, both of which are the main reasons for cirrhosis [23, 24]. Additionally, animal experiments discovered that inhibition of PCSK9 could promote LDL-R expression, thus protecting cirrhosis rats from intestinal origin endotoxemia, a common complication in decompensate cirrhosis that results in high mortality [88]. Thus, PCSK9 inhibitors may exert multiple beneficial effects on decompensated cirrhosis.

Nevertheless, serum PCSK9s were found to increase during the liver fibrosis progress but decrease significantly in the terminal stage of cirrhosis, possibly due to severely impaired liver function resulting in reduced PCSK9 synthesis [89, 90]. This phenomenon might weaken the efficiency of PCSK9 inhibitors in decompensated cirrhosis patients. Coupled with a lack of clinical data among Child-Pugh C patients, the efficiency and safety of PCSK9 inhibitors’ application in patients with decompensated cirrhosis requires further study.

### 5.3 Renal Dysfunction

#### 5.3.1 Relationship with the Kidney

The comparison of statins and PCSK9 inhibitors in the relationship with the kidney is listed in Table 4.

**Table 4.** **Comparison of statins and PCSK9 inhibitors in the relationship with the kidney**.

Relationship with the kidney	Statins	PCSK9 inhibitors
Renal elimination	YES	NO
Proteinuria	Induce proteinuria	Do not induce proteinuria
eGFR	Cause decreases in eGFR	Have no effect on eGFR
AKI	YES	YES

eGFR, estimated glomerular filtration rate; AKI, acute kidney injury; PCSK9, proprotein convertase subtilisin/kexin type 9.

5.3.1.1 Statins

*In vivo*, statins are eliminated by the kidney in a small percentage (<20%) [20]. Impaired renal function can result in higher systemic statin exposure, leading to a higher risk of statin-induced AEs (OR = 1.20; *p *< 0.009) of SI [33]. Therefore, a nearly 50% adjustment of various statin doses in patients with advanced chronic kidney disease (CKD) (eGFR <60 mL/min/1.73 $m^{2}$) is recommended by the Kidney Disease: Improving Global Outcomes (KDIGO) guideline [91].

On the other hand, statins themselves have also been confirmed to have a certain degree ossf nephrotoxicity. Statin-induced AKI has been previously discussed in the AKI section. Evidence suggests that statins may induce proteinuria and cause decreases in eGFR [92]. A large-scale retrospective cohort study involving 68256 patients under statin treatment demonstrated that high-efficiency statin treatment was associated with a 13% increased risk (HR = 1.13; [95% CI: 1.02–1.26]) of developing severe renal failure (defined as in need of renal replacement therapy) compared with low-efficiency statins. A dose-dependent effect was discovered between the cumulative dose of statins and severe renal failure [93].

5.3.1.2 PCSK9 Inhibitors

Current FDA-approved PCSK9 inhibitors are mAbs and do not undergo renal elimination [27]. Hence, PCSK9 inhibitors do not require dose adjustment in the renal insufficiency population [16, 17]. Nephrotoxicity of PCSK9 inhibitors has been rarely reported. A prespecified analysis of the ODYSSEY OUTCOMES trial, including 18,924 patients focusing on the efficiency and safety of alirocumab in patients with diverse renal function, revealed a similar AEs incidence among all eGFR subgroups, and alirocumab showed no effect on eGFR (*p* = 0.65) during a follow-up period of 36 months [94]. Although PCSK9 is expressed in the renal tissue, serum PCSK9 levels do not appear to be associated with renal function [95].

Given concerns about statins’ increased risk of AEs and non-negligible nephrotoxicity in renal insufficiency patients, a growing number of researchers are interested in using PCSK9 inhibitors in patients with renal dysfunction.

#### 5.3.2 Dialysis

5.3.2.1 Statin Status

Dyslipidemia occurs in up to 82% of dialysis patients. The CV risk for patients with CKD receiving dialysis is 20 times that of the general population [95, 96].

According to current guidelines, statins are not recommended in dialysis-dependent CKD patients because of efficacy and safety concerns [2, 3, 91]. This conclusion is mostly based on the 4D (Deutsche Diabetes Dialyze Studie) [97] and the AURORA (An Assessment of Survival and Cardiovascular Events) trials [98], demonstrating that statins afford no improvement on CV outcomes and all-cause mortality in patients on dialysis.

Previous studies attribute this lack of benefits to the different CV disease pathophysiologies in patients on dialysis [20]. Declining renal function is associated with metabolic disturbances, inflammation, and impaired mineral homeostasis, resulting in accelerated atherosclerosis and vascular calcification, which are non-modifiable by delayed statin initiation [99]. Therefore, the efficiency of statins diminishes with the decrease in eGFR and shows no benefit in dialysis-dependent kidney disease. The procalcifying effect of statins may accelerate vascular calcification in patients on dialysis and increase the risk of adverse CV events [20]. The increased risk of statin-induced AEs [30] make statins an unsatisfactory lipid-lowering option in CKD patients on dialysis.

5.3.2.2 Safety of PCSK9 Inhibitors in Dialysis

Unlike statins, which showed a diminishing protective effect with a decrease in eGFR, the safety and efficiency of PCSK9 inhibitors remain stable in patients with varying renal function [94, 95, 100].

Charytan DM *et al*. [100] performed a subgroup analysis of the FOURIER trial to investigate the efficiency and safety of evolocumab according to renal function. The primary endpoint was a composite of CV death, myocardial infarction (MI), stroke, coronary revascularization, and hospitalization for unstable angina. The secondary endpoint was a composite of CV death, MI, and stroke. The study demonstrated infrequent and similar AEs rates among all CKD stage groups. More importantly, it revealed a similar and significant reduction in the relative risk of the primary endpoints (HR = 0.82; [95% CI: 0.71–0.94] for preserved renal function, HR = 0.85; [95% CI: 0.77–0.94] for stage 2 CKD, and HR = 0.89; [95% CI: 0.76–1.05] for stage ≥3 CKD) and secondary endpoints (HR = 0.75; [95% CI: 0.62–0.90] for preserved renal function, HR = 0.82; [95% CI: 0.72–0.93] for stage 2 CKD; and HR = 0.79; [95% CI: 0.65–0.95] for stage ≥3 CKD) among all CKD stage groups [100]. Since worse renal function is associated with higher event rates, the absolute reduction in ASCVD events has greater clinical significance in advanced CKD.

In CKD patients on dialysis, the protein loss enhances Lp (a) liver synthesis and elevated Lp (a) concentration [101]. This atherogenic lipoprotein can increase CV morbidity and mortality. PCSK9 inhibitor is the only approved lipid-lowering drug that can significantly reduce Lp (a) level by 30–40% [3]. Hence PCSK9 inhibitors may bring extra lipid-lowering benefits to patients on dialysis [101].

The favorable effect of PCSK9 inhibitors’ on eGFR is undoubtedly an essential advantage compared with the poor performance of statins. Nevertheless, it should be noted that individuals with eGFR <20 mL/min/1.73 $m^{2}$ (most of which required dialysis) were excluded in the FOURIER trial [100], and there is a lack of data on the application of PCSK9 inhibitors in patients on dialysis. Large-scale studies on dialysis patients are warranted to confirm these results in comparison with statins.

## 6. Safety of PCSK9 Inhibitors with Statin-Interactive Drugs

Drug-drug interaction (DDI) has become one of the leading causes of drug restriction and poor adherence, occurring in up to 50% of the elderly population (32.6% of men and 49.2% of women) [102].

The pharmacokinetics of statins and PCSK9 inhibitors have been described in the Clinical Pharmacology section. The mechanisms of most statin-drug interactions are competitive inhibition of the CYP450 isoenzymes (mainly CYP3A4) or drug transporters (mainly OATP1B1 and P-gp) [103]. The inhibition of statins’ metabolism or elimination pathway increases plasma statin AUC and Cmax, leading to a higher risk of severe statin-induced AEs, such as fatal rhabdomyolysis and acute renal failure [18].

A large number of drugs have reported DDI with statins [18, 103]. Our review is not intended to give a comprehensive list but focuses on drugs specifically used for certain diseases that have intensive DDIs with statins. These including anti-HIV, anti-HCV, and immunosuppressive drugs.

**Fig. 3.**
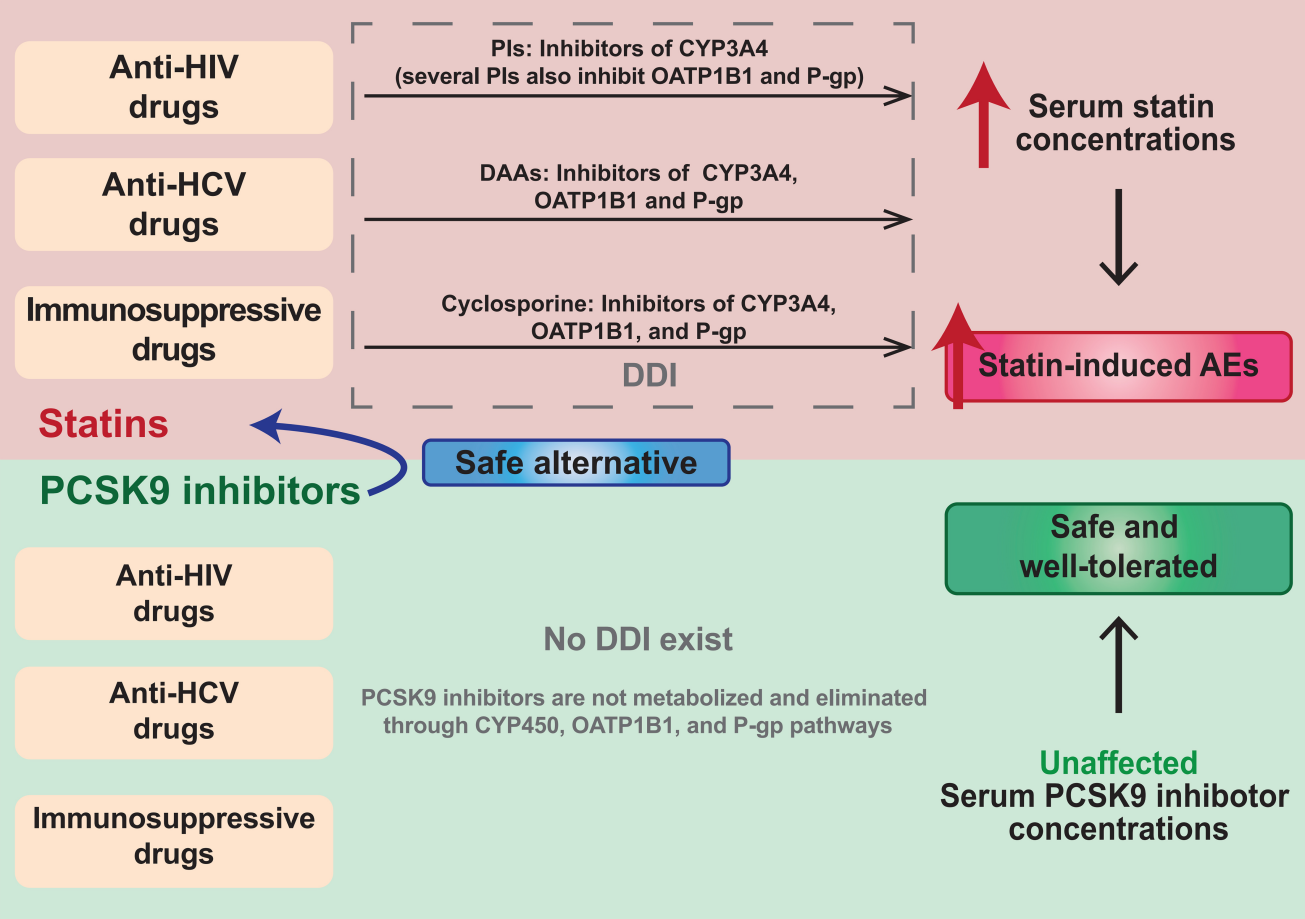
**Safety comparisons between statins and PCSK9 inhibitors when coadministered with anti-HIV, anti-HCV, and immunosuppressive drugs**. PI, protease inhibitor; CYP3A4, cytochrome P450 3A4; OATP1B1, organic anion transporting polypeptide 1B1; P-gp, P-glycoprotein; DDI, drug-drug interaction; CYP450, cytochrome P450.

Current FDA-approved PCSK9 inhibitors are mAbs and thus are not affected by the CYP450 isoenzyme system, OATP1B1, and P-gp pathways that metabolize and eliminate statins. Hence, PCSK9 inhibitors are ideal for alternative non-statin LLT in HIV, HCV, and solid organ transplantation patients. The safety comparisons between statins and PCSK9 inhibitors when coadministered with anti-HIV, anti-HCV, and immunosuppressive drugs are summarized in Fig. 3.

### 6.1 Anti-Human Immunodeficiency Virus (HIV) Drugs

#### 6.1.1 Statin Status

Type I HIV infection can lead to lipid metabolism disorders and chronic inflammation, causing a significantly higher risk of dyslipidemia (67.3% in women and 81.2% in men) among HIV-infected patients and resulting in a higher incidence of ASCVD [104].

Nevertheless, statins are widely restricted or abandoned in patients with HIV infection [105]. On March 1st, 2012, The FDA updated a safety announcement on statins, warning about the DDIs between HIV protease inhibitors and statins [18]. Among numerous HIV-infected patients with dyslipidemia, only 5.9% acquired statin therapy [106].

There are complex interactions between statins and antiretroviral drugs. On the one hand, protease inhibitors (PIs), the first class of antiretroviral therapy in HIV-infected patients, are strong CYP3A4 inhibitors. Several PIs are also inhibitors of OATP1B1 and P-gp. Consequently, the DDIs between PIs and statins are intensive and involve multiple pathways [105]. A 30-fold increase (*p *< 0.001) in the AUC of simvastatin was observed in patients taking saquinavir and ritonavir in the ACTG (AIDS Clinical Trials Group) A5047 study [107]. Cases of rhabdomyolysis have been reported in patients receiving both statins and PIs.

On the other hand, conversely, the non-nucleoside reverse transcriptase inhibitors, such as nevirapine, efavirenz, and etravirine, are CYP3A4 isozymes inducers, diminishing the efficiency of statins when given simultaneously [97]. The ACTG A5108 study, which included 52 HIV-infected patients, demonstrated a 60% (*p* = 0.003), 50% (*p *< 0.001), and 40% (*p* = 0.005) decrease in the AUC of simvastatin, atorvastatin, and pravastatin when coadministered with efavirenz, respectively [108].

#### 6.1.2 Safety of PCSK9 Inhibitors with Anti-HIV Drugs

The metabolism and elimination of PCSK9 inhibitors are independent from the CYP450 isoenzyme system and OATP1B1 or P-gp drug transporters. No DDI has been discovered between PCSK9 inhibitors and anti-HIV drugs.

The EvolocumaB Effect on LDL-C Lowering in SubJEcts with Human Immunodeficiency VirRus and INcreased Cardiovascular RisK (BEIJERINCK) study was the first study assessing the safety and efficiency of evolocumab treatment in 464 patients under anti-HIV therapy during a 24-week follow-up period. After adjusting confounder factors, evolocumab and placebo groups showed similar treatment-emergent AEs and serious AEs (67.5% versus 61.9% and 5.1% versus 3.3%, respectively). Evolocumab also showed a consistent lipid-lowering effect despite well-performed tolerability, lowering LDL-C levels by 56.9% (95% CI: 61.6–52.3%), unaffected by antiretroviral therapy [109].

In addition to their safety and efficacy, PCSK9 inhibitors are expected to provide additional therapeutic benefits in HIV-infected patients. PCSK9 levels are 65% higher (*p *< 0.0001) in HIV-infected patients than in uninfected patients and are associated with decreased coronary endothelial dysfunction (R = –0.51; *p *< 0.0001) in a cross-sectional study including 48 patients [25]. The anti-inflammatory effects of PCSK9 inhibitors are thought to suppress the HIV-induced chronic inflammatory state [25].

Nevertheless, there are too few existing studies to determine the safety of PCSK9 inhibitors in HIV-infected patients, and more data is warranted.

### 6.2 Anti-HCV Drugs

#### 6.2.1 Statin Status

Chronic hepatitis C virus (HCV) infection is an independent risk factor for CVD. However, only 21% of HCV infected patients who meet lipid-lowering standards received statin therapy [110].

The main reason is that statins are strictly limited in HCV-infected patients due to concerns about DDIs with anti-HCV drugs [18, 105].

Direct-acting antiviral agents (DAAs) act as the most effective anti-HCV therapy, significantly improving clinical treatment success rates to 95%. Unfortunately, many DAAs are inhibitors of CYP3A4enzymes, OATP1B1, and P-gp [83]. Patients who use DAAs in combination with statins have a 1.5–10 fold increase in statin plasma concentrations and an increase in the risk of statin-induced AEs [83, 111]. According to a large prospective cohort study in Italy analyzing 814 patients who underwent DAAs, statins rank as one of the most frequently (accounting for 20–35% of patients) suspended or replaced drugs due to DDIs [112].

Coadministration of statins and DAAS are not recommended because of the increased incidence of DDIs. Only the lowest statin doses are permitted, along with careful monitoring for myopathy/rhabdomyolysis [18, 83].

#### 6.2.2 Safety of PCSK9 Inhibitors with Anti-HCV Drugs

PCSK9 inhibitors are not metabolized by the CYP450 isoenzymes system or OATP1B1 and P-gp drug transporters. No case of DDI with DAAs has been reported [111].

Dousdampanis, Assimakopoulos *et al*. [113] reported that in a male patient on hemodialysis with dyslipidemia and chronic HCV infection comorbidities, atorvastatin was discontinued because of the concern for hepatotoxicity. Alirocumab was prescribed as an alternative option (150 mg every 2 weeks, 8 weeks in total), and a significant decrease in LDL-C levels was observed without side effects [113].

In addition to concerns about DDIs, impaired hepatic function in HIV-infected patients is another important reason for limiting statins. As discussed in the Liver dysfunction section, PCSK9 inhibitors are safe in patients with hepatic impairment, including HIV-infected patients [83, 87]. HCV infection is associated with high PCSK9 levels *in vivo * [114, 115]. Although some studies propose that PCSK9 plays a protective inhibitory role in HCV infection by enhancing LDL receptor degradation and downgrading CD81 expression (both are receptors that mediate HCV entry into hepatocytes) [116], the evidence from *in vitro* and *in vivo* studies demonstrated that inhibition of PCSK9 neither effect CD81 levels nor increase susceptibility to HCV entry [114, 117].

In summary, PCSK9 inhibitors are a potential alternative to statins in HCV-infected patients for two main reasons: (1) Freedom from interactions with HCV medications and (2) a more favorable liver safety profile. Future studies investigating the use of PCSK9 inhibitors in large cohorts of different degrees of hepatic impairment and virus loads are necessary to further clarify the safety and tolerability of PCSK9 inhibitors in HCV-infected patients.

### 6.3 Immunosuppressive Drugs

#### 6.3.1 Statins Status

Solid-organ transplantation (SOT) recipients have a high incidence of dyslipidemia (80% in kidney transplant recipients, over 50% in heart transplant recipients, and 30–50% in liver transplant recipients), which contributes to increased CVD events, which is one of the leading causes of mortality [118]. Hence, guidelines have placed great emphasis on LLTs, requiring LLTs as part of standard post-transplant therapies regardless of cholesterol levels [2, 3, 119].

However, statin use among SOT recipients is limited because of DDIs associated with immunosuppressive drugs [18].

Immunosuppressive drugs, especially cyclosporine, have been shown to interact with statins. Cyclosporine is an inhibitor of multiple pathways in statin metabolism and elimination (including CYP3A4, OATP1B1, and P-gp pathways). Hence, there is a strong DDI between cyclosporine and statins [120]. Cyclosporine can increase the AUC of atorvastatin and simvastatin by 7.5-fold and 8-fold, respectively [121]. Cases of rhabdomyolysis have been reported in patients receiving both statins and cyclosporines [122]. Therefore, most statins are not recommended a with cyclosporines [120].

Besides statins, other lipid-lowering drugs such as ezetimibe, gemfibrozil, and fenofibrate have also been associated with DDIs with immunosuppressive drugs, resulting in increased AEs [118].

#### 6.3.2 Safety of PCSK9 Inhibitors with Immunosuppressive Drugs

Enthusiasm has grown for PCSK9 inhibitors as a safer alternative LLT in SOT recipients who have failed to tolerate statins.

A clinical case series involving different kinds of SOT (including heart, kidney, liver, and lung transplantation) reported no severe AEs or discontinuation in 25 participants receiving PCSK9 inhibitors (evolocumab and alirocumab) and various immunosuppressive drugs (including cyclosporine and tacrolimus) coadministration [123]. Jennings, Jackson, *et al*. [124] identified 5 heart transplant recipients who receive PCSK9 inhibitor therapy (evolocumab 140 mg every two weeks) because of statin-induced myositis. The duration of evolocumab ranged from 12–34 months and no AE was noted.

The Cholesterol lowering with EVOLocumab to prevent cardiac allograft Vasculopathy in De-novo heart transplant recipients (EVOLVD) trial (NCT03734211) is an ongoing double-blind, randomized, placebo-controlled trial investigating the effect of evolocumab on cardiac allograft vasculopathy among heart transplant recipients [125]. This trial will further determine the feasibility of PCSK9 inhibitors as an alternative LLT in SOT patients.

## 7. Discussion

Statins act as the first-line therapy of current lipid management strategy. Nevertheless, statin use is limited in many conditions, and there are gaps in lipid-lowering regimens in certain high risk patient populations that need to be filled.

PCSK9 inhibitors have been validated in large clinical trials as a promising new drug with excellent lipid-lowering effects and general tolerability. However, current guidelines only conservatively recommend PCSK9 inhibitors as an add-on therapy to maximumly tolerated statin and ezetimibe therapy or an alternate in patients with SI (essentially SAMS) [2, 3]. The clinical use of PCSK9 inhibitors is critically limited. Although a growing number of studies in recent years have focused on and confirmed the safety of PCSK9 inhibitors in different categories of patients, there is a lack of clinical studies and reviews that systematically answer the questions raised by the 2018 ACC/AHA and 2019 ESC/EAS guidelines [2, 3]: Are PCSK9 inhibitors a safe alternative lipid-lowering therapy in patients whose statin use is limited?

**Fig. 4.**
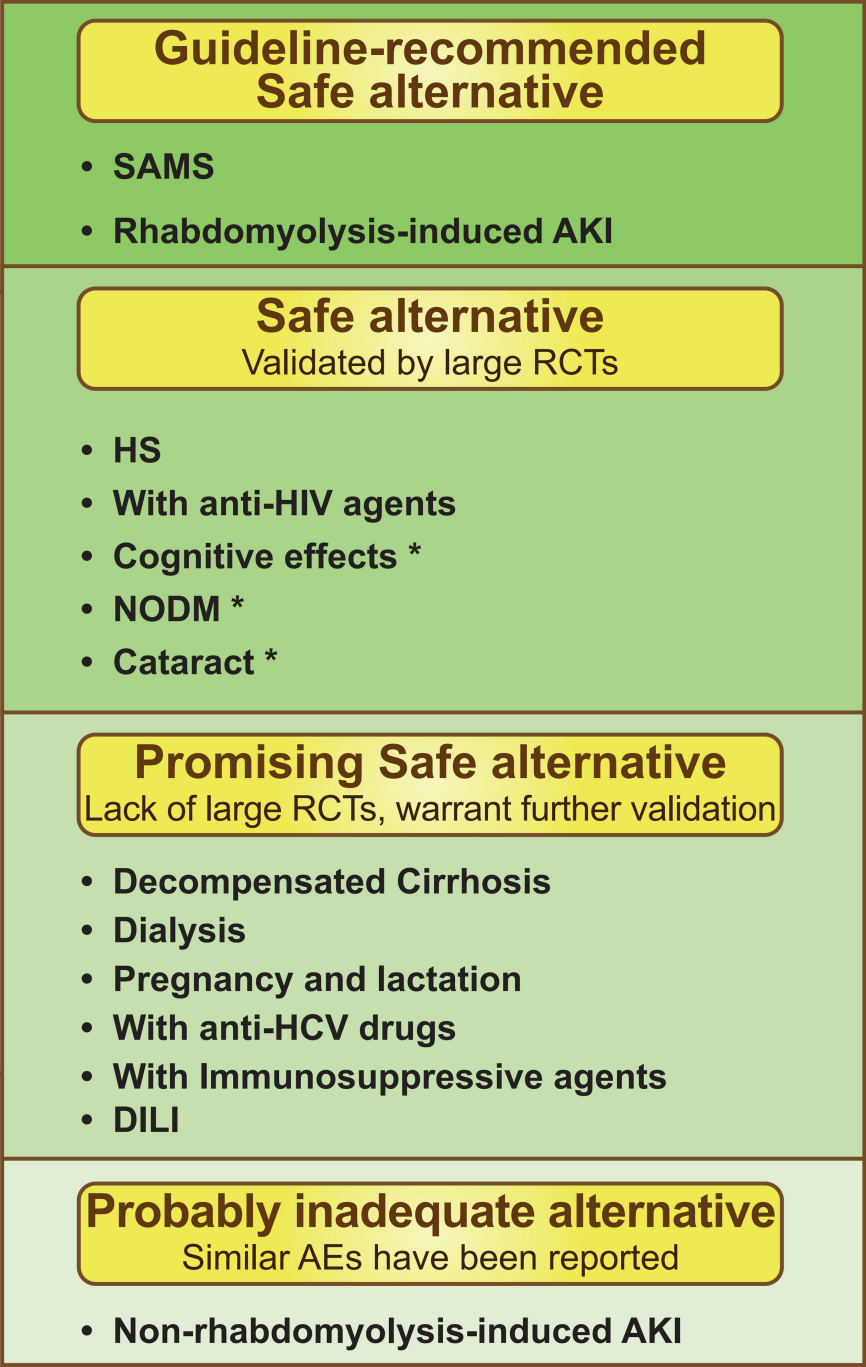
**Overview of the safety of PCSK9 inhibitors as statin alternative therapy in statin-limited conditions**. SAMS, statin-associated muscle symptoms; AKI, acute kidney injury; HS, hemorrhagic stroke; HIV, human immunodeficiency virus; NODM, new-onset diabetes mellitus; HCV, hepatitis C virus; DILI, drug-induced liver injury. * Warrant further analyses to find the beneficiaries of PCSK9 inhibitors regarding the mild degree of statin-induced AEs or controversial relationship with statins.

This review concluded that PCSK9 inhibitors show good safety profiles in various statin-limited conditions (Fig. 4). First, PCSK9 inhibitors are already recognized in the guidelines as alternative therapy in patients with SAMS, including rhabdomyolysis-induced AKI [2, 3, 59]. Second, PCSK9 inhibitors have been shown in large RCTs not to induce NODM, HS, cognitive effects, and cataracts, and do not have DDIs with anti-HIV drugs [21, 47, 48, 66, 74, 109]. Hence, PCSK9 inhibitors are a safe alternative in the above conditions. Nevertheless, due to the mild degree of statin-induced AEs, statin use is not strictly limited in patients at risk for NODM, cognitive impairment, and cataracts [18, 45, 71]. Hence, further careful analyses are warranted to determine which patients will benefit from PCSK9 inhibitors. Third, PCSK9 inhibitors have been found to be safe alternatives in DILI, pregnancy and lactation, decompensated cirrhosis, dialysis, and coadministration with anti-HCV and immunosuppressive drugs in current studies [16, 17, 53, 81, 113, 124]. Investigations of the application of PCSK9 inhibitors in these conditions are still in the preliminary stages, so large clinical trials are somewhat limited to date. Further studies are necessary to confirm the safety of PCSK9 inhibitors’ in these patient populations. Finally, for patients with non-rhabdomyolysis-induced AKI, a cautious attitude should be adopted in considering PCSK9 inhibitors as statin alternative therapy, given the presence of, rare, case reports of PCSK9 inhibitor-induced AKI [60, 61].

Several factors limit us from drawing further conclusions: First, many safety outcomes were conducted by trials investigating PCSK9 inhibitors as add-on therapy with statins, or statins and ezetimibe rather than monotherapy. Second, large clinical trials of PCSK9 inhibitors generally have the control group as a placebo or ezetimibe, and lack a direct comparison with the safety of statins. Third, current safety conclusions of PCSK9 inhibitors are based on a relatively short follow-up period (the longest 4 years) [15]. Although the preliminary results are quite promising, previous experience from other studies have found that trials with longer follow-up periods are necessary to confirm its long-term safety. Fourth, there is a lack of studies within statin-limited subgroups with risk-benefit comparisons between statins and PCSK9 inhibitors to identify those patients who will benefit most from PCSK9 inhibitors.

Another issue that limits the widespread use of PCSK9 inhibitors is its high cost. However, a 60% discount (approximately $5850) was recently announced by manufacturers of alirocumab and evolocumab in 2018 and 2019 [126]. Moreover, several more cost-effective new PCSK9 inhibitors, such as the small interfering RNA inclisiran and PCSK9 vaccines, are under development [127]. It is expected that PCSK9 inhibitors will have a prominent role in lipid management strategy in the foreseeable future.

In conclusion, our review provides the first comprehensive discussion of the safety of PCSK9 inhibitors in different statin-limited patient populations, showing that PCSK9 inhibitors is a safe and efficacious alternative to statins in these patients, paving the way for future research.

## 8. Conclusions

We sought to determine if PCSK9 inhibitors are a safe alternative to statins in patients with statin-limited conditions. We therefore performed a thorough review on the safety of PCSK9 inhibitors in statin-limited conditions (including statin-induced AEs, special populations, and DDIs) from pharmacological, epidemiological and clinical aspects. We concluded that PCSK9 inhibitors are the guideline-recommended alternative therapy for SAMS, including rhabdomyolysis-induced AKI. PCSK9 inhibitors are a safe alternative to statins, do not induce NODM, HS, cognitive effects, and cataracts, and do not have DDIs with anti-HIV drugs. Further careful analyses are warranted to find the beneficiaries of PCSK9 inhibitors in patients at risk for NODM, cognitive impairments, and cataracts. PCSK9 inhibitors are a promising alternative in DILI, pregnancy and lactation, decompensated cirrhosis, dialysis, and with anti-HCV and immunosuppressive drugs. However, large RCTs are lacking in these populations, and further clinical studies are necessary to further confirm the safety of PCSK9 inhibitors. PCSK9 inhibitors are probably an inadequate alternative for non-rhabdomyolysis-induced AKI, based on the existing case reports. Furthermore, we identified an insufficiency in the current research and indicated future areas for investigation. The safety of PCSK9 inhibitors should be confirmed in large, adequately powered long-term, monotherapy, statin-controlled trials with careful cost-effectiveness analyses.

## Abbreviations

CV, Cardiovascular; ASCVD, atherosclerotic cardiovascular disease; LDL-C, low-density lipoprotein cholesterol; AHA, American Heart Association; AE, adverse event; LLT, lipid-lowering therapy; PCSK9, proprotein convertase subtilisin/kexin type 9; FDA, the US Food and Drug Administration; CVD, cardiovascular disease; HMG-CoA, 3-hydroxy-3-methylglutaryl–coenzyme A; TG, triglyceride; HDL-C, high-density lipoprotein cholesterol; OATP1B1, organic anion transporting polypeptide 1B1; OATP1B3, organic anion transporting polypeptide 1B3; CYP450, cytochrome P450; CYP3A4, cytochrome P450 3A4; CYP2C9, cytochrome P450 2C9; P-gp, P-glycoprotein; IgG, immunoglobulin G; mAb, monoclonal antibody; Lp (a), lipoprotein a; SAMS, statin-associated muscle symptoms; SI, statin intolerance; OR, odds ratio; 95% CI, 95% confidence interval; HR, hazard ratio; RR, relative risk; NODM, new-onset diabetes mellitus; T2D, type 2 diabetes; DILI, drug-induced liver injury; AKI, acute kidney injury; HS, hemorrhagic stroke; ALT, alaninetransaminase; AST, aspartate transaminase; CKD, chronic kidney disease; eGFR, estimated glomerular filtration rate; DDI, drug-drug interaction; AUC, area under curve; Cmax, maximum plasma concentration; HCV, hepatitis C virus; DAA, direct-acting antiviral agent; HIV, anti-human immunodeficiency virus; PI, protease inhibitor; SOT, solid-organ transplantation.
